# A Rare Case of a Forgotten Common Bile Duct Stent Retained for 23 Years

**DOI:** 10.7759/cureus.114373

**Published:** 2026-08-11

**Authors:** Manish Kumar, Anchal Kumari, Chongtham Anil Kumar Singh, Ajay Dhiman, Mohim Thakur

**Affiliations:** 1 Department of General Surgery, All India Institute of Medical Sciences (AIIMS), Bilaspur, IND

**Keywords:** cbd explorations, cholangitis, choledochoduodenostomy, forgotten cbd stent, giant stentolith

## Abstract

Forgotten biliary stents are rarely encountered in hepatobiliary practice. Most patients undergo timely removal of a common bile duct (CBD) stent by endoscopic retrograde cholangiopancreatography (ERCP), or sometimes the stent may spontaneously migrate into the gastrointestinal tract and be expelled with stool. Delayed removal or prolonged retention of plastic CBD stents increases the risk of cholangitis, stent blockage, and stent stone formation. We report a case of a 54-year-old male patient with a forgotten stent for almost 23 years, with recurrent episodes of cholangitis and hospital admissions. The patient was initially managed for cholangitis and subsequently underwent open CBD exploration with removal of the stent-stone complex, followed by side-to-side choledochoduodenostomy. This case highlights the importance of follow-up, proper patient communication, and counseling regarding the importance of timely removal of CBD stents to avoid complications related to forgotten stents.

## Introduction

Common bile duct (CBD) stenting is one of the established interventions used to relieve biliary obstruction caused by conditions like choledocholithiasis, biliary strictures, and hepatobiliary cancers [1]. Conditions requiring temporary drainage of the CBD are managed with plastic stents, whereas conditions requiring long-term drainage, such as palliative drainage, poor surgical candidacy, or patients with significant comorbidities, are managed with metallic stents [2,3]. A retained CBD stent may result in complications like recurrent cholangitis, biliary stone formation, CBD stricture, and secondary biliary cirrhosis [1,4,5]. Many cases of long-term CBD stent retention have been reported, but CBD stent retention for two decades is exceedingly rare. We present a case of a forgotten CBD stent retained for 23 years, resulting in recurrent hospital admissions due to cholangitis, which was subsequently managed with open CBD exploration and choledochoduodenostomy.

## Case presentation

A 54-year-old male presented with recurrent episodes of fever, right upper abdominal pain, and jaundice over the last three years. The pain was intermittent and colicky in nature and was associated with nausea. The patient reported a history of endoscopic retrograde cholangiopancreatography (ERCP) with biliary stent placement for choledocholithiasis 23 years earlier in 2002. The patient also gave a history of open cholecystectomy one year after the ERCP. Following the procedure, the patient remained asymptomatic for many years. However, over the past three years, he developed similar complaints and required multiple hospital admissions. No documentation regarding stent removal or follow-up was available. On examination, the patient was febrile and icteric, with tenderness in the right hypochondrium. Laboratory investigations revealed leukocytosis, cholestatic liver function elevations, and deranged renal function tests. A full picture of the results is presented in Table 1.

**Table 1:** Comparison of laboratory investigations at admission and six-month follow-up after surgery.

Laboratory parameter	Admission	6-month follow-up after surgery	Reference range
Hemoglobin (g/dL)	11.3	13.5	13-16
Total leukocyte count (×10³/µL)	18.8	5.5	4-12
Total bilirubin (mg/dL)	5.1	0.9	0.2-1.2
Direct bilirubin (mg/dL)	4.1	0.3	0.0-0.3
Alkaline phosphatase (ALP) (U/L)	900	130	40-130
Aspartate aminotransferase (AST) (U/L)	132	48	10-40
Alanine aminotransferase (ALT) (U/L)	148	36	10-55
Gamma-glutamyl transferase (GGT) (U/L)	312	56	15-85
Serum albumin (g/dL)	3	3.5	3.5-5
C-reactive protein (CRP) (mg/L)	150	4	<5
Blood urea nitrogen (mg/dL)	60	20	7-20
Serum creatinine (mg/dL)	1.9	1.1	0.7-1.3
Prothrombin time (s)	18	12.6	11-14
International normalized ratio (INR)	1.6	1	0.8-1.2

**Figure 1:**
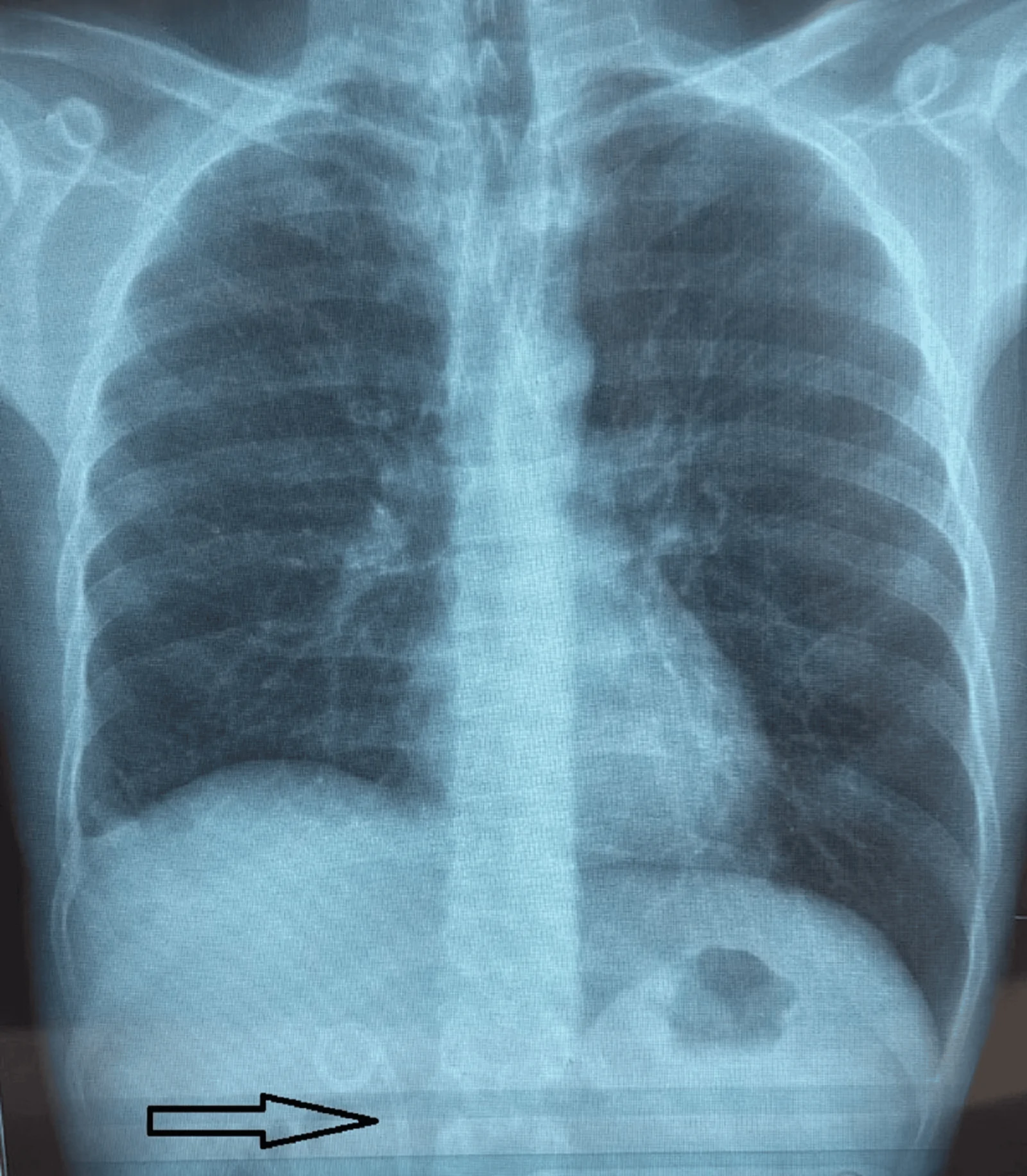
X-ray showing CBD stent in situ (black arrow). CBD: common bile duct

Blood culture was suggestive of *E. coli *bacteremia. Ultrasonography (USG) demonstrated dilated intrahepatic biliary radicals and a dilated CBD containing echogenic material. The gallbladder was not visualized on ultrasound. A chest radiograph revealed a radiopaque structure suggestive of a retained biliary stent (Figure 1).

**Figure 2:**
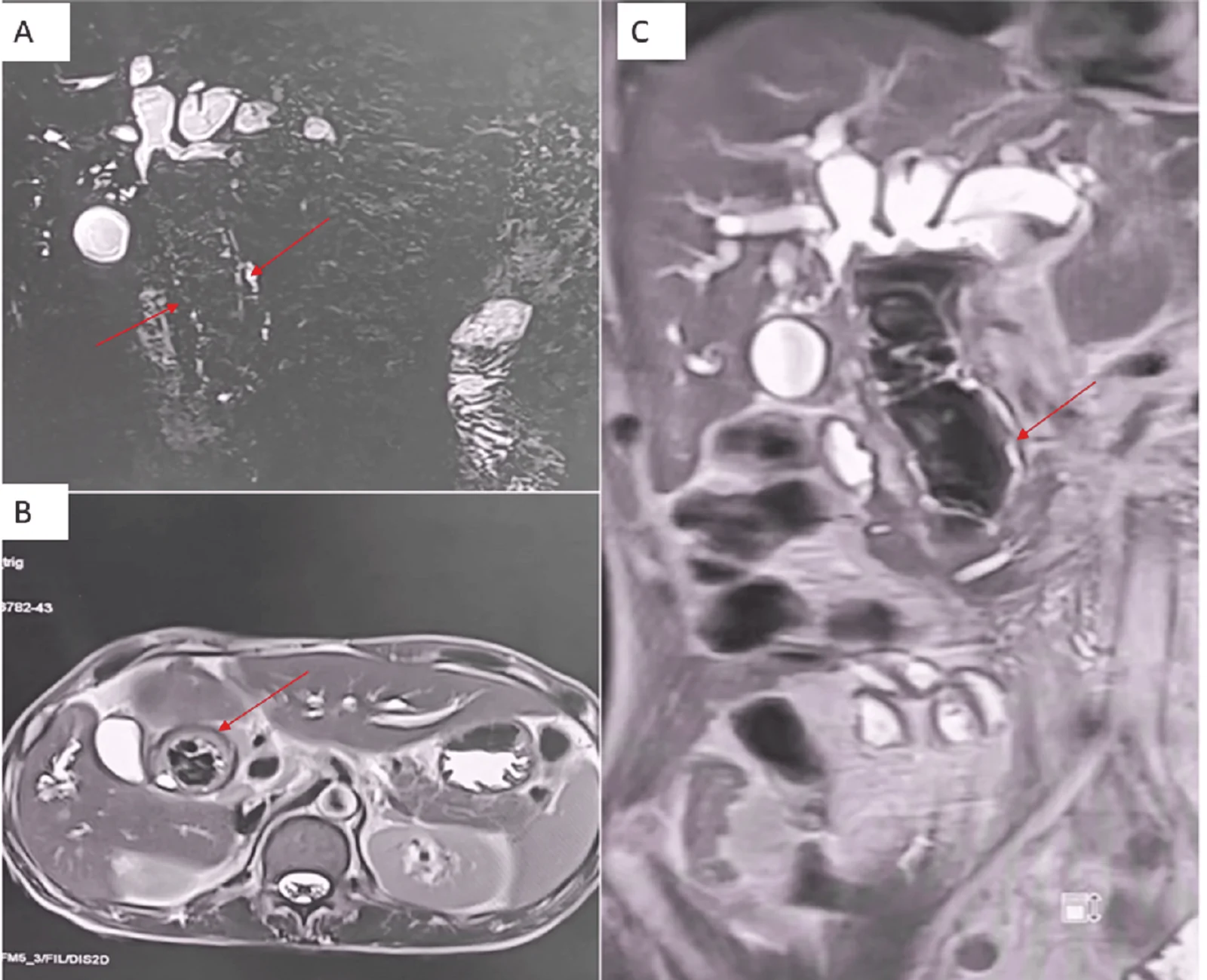
Magnetic resonance cholangiopancreatography (MRCP) images. (A) Heavily T2-weighted MRCP image showing a linear intraductal signal void within the common bile duct (red arrows) with surrounding intraductal filling defect. (B) Axial T2-weighted image demonstrating a rounded hypointense intraluminal lesion within a dilated CBD (red arrow). (C) Coronal MRCP image showing CBD dilatation with a central linear signal void and adherent filling defect (red arrow), causing biliary obstruction. CBD: common bile duct

Magnetic resonance cholangiopancreatography (MRCP) showed a markedly dilated CBD measuring 28 mm, containing T2-hypointense material extending along its entire length up to the duodenal papilla, measuring approximately 2.2×2.5×8.3 cm (anteroposterior×transverse×craniocaudal). Bilobar intrahepatic biliary dilatation with a patent primary confluence was noted (Figures 2A-2C).

**Figure 3:**
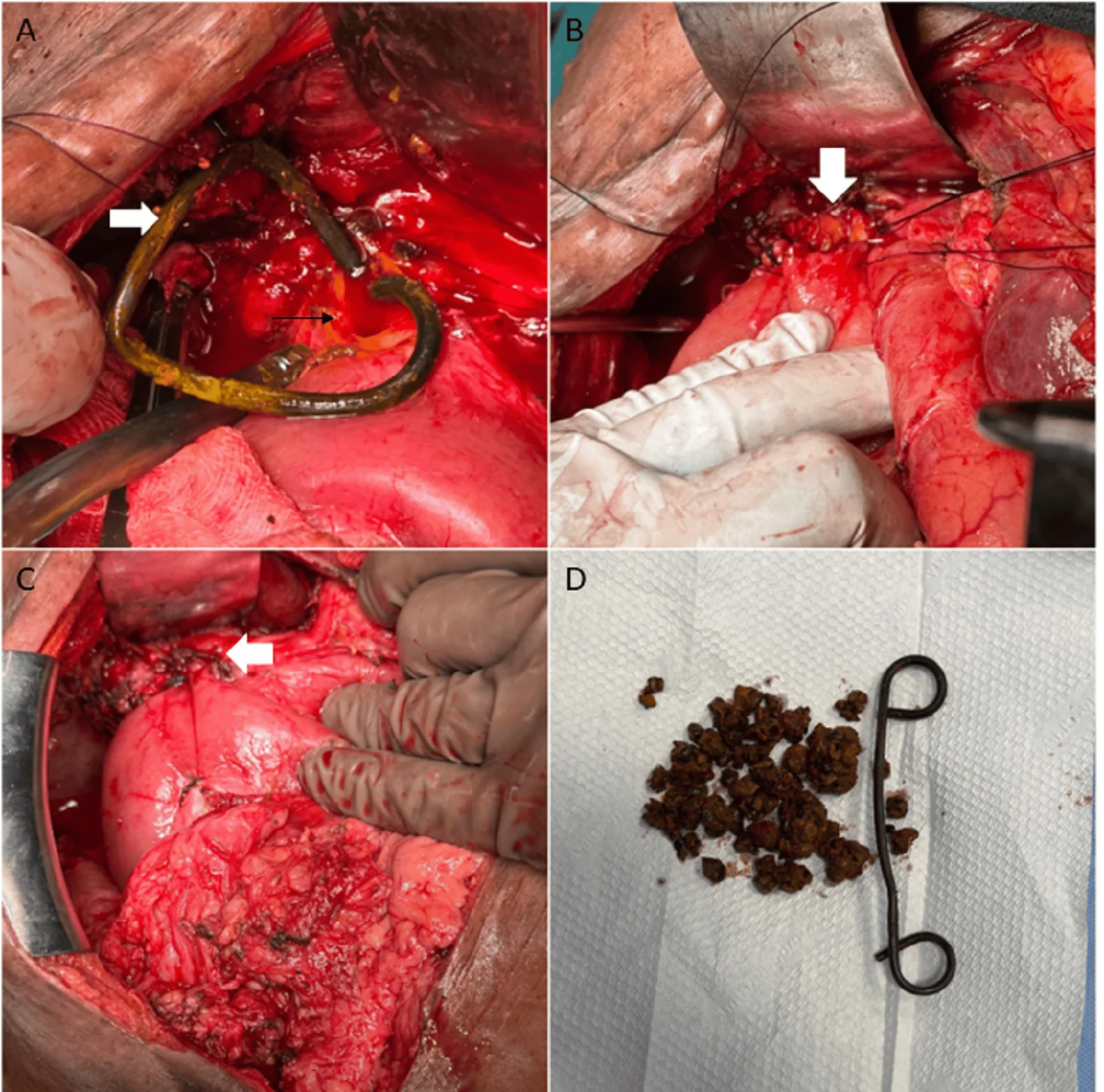
Intraoperative images of retrieved plastic biliary stent with heavy encrustation and multiple CBD calculi. (A) Choledochotomy site (black arrow) and retrieval of retained stent (white arrow). (B, C) Choledochoduodenostomy anastomosis (white arrow). (D) Retained stent along with stones. CBD: common bile duct

The residual gallbladder was contracted without evidence of calculi. Given the prolonged duration of stent retention, recurrent cholangitis, and significant stone burden, the patient was optimized and treated conservatively for cholangitis prior to definitive management. He subsequently underwent an open cholecystectomy with CBD exploration. Intraoperatively, the CBD was found to be markedly dilated, inflamed, and dense adhesions were present in the subhepatic area. After meticulous dissection, the CBD was identified, and a longitudinal choledochotomy was performed to access the ductal lumen. A plastic biliary stent, heavily encrusted with biliary sludge and stones, was retrieved along with multiple CBD calculi (Figures 3A-3D).

Following complete ductal clearance, a side-to-side choledochoduodenostomy was performed to ensure adequate biliary drainage. The postoperative course was uneventful. The patient showed marked clinical improvement, with resolution of jaundice and improvement of liver function parameters. He was discharged in stable condition on postoperative day seven and remained asymptomatic on follow-up. At six-month follow-up, laboratory parameters showed improvement following surgery (Table 1).

## Discussion

Choledocholithiasis affects 10-15% of patients with gallstone disease. Most cases are managed with ERCP. Plastic stent placement is done following CBD stone clearance for temporary drainage. These plastic stents have short patency duration, increase the risk of cholangitis and stone formation, and therefore require frequent removal or exchange compared to metallic stents [6,7]. When these stents are retained in CBD for more than one year, they are termed forgotten stents [1,3,8]. Prolonged stent retention promotes sludge accumulation and bacterial colonization, which can precipitate recurrent biliary infections [1,3,5-8]. Previous studies have demonstrated that the main contributing factor for retained stents is poor compliance with follow-up visits. The various reasons for poor compliance are immediate symptom relief after stenting, poor documentation in the discharge card, and lack of proper communication and counseling of patients and attendants [2,3,5]. Forgotten CBD stents are a rare condition, as the majority of plastic CBD stents are removed within three months. Elsharif et al. reported a 14-year period of forgotten CBD stent retention in 2025 [8]. In the present case, the stent had remained in situ for 23 years (276 months) and was associated with recurrent episodes of cholangitis and stentolith formation, highlighting the potential long-term complications of prolonged biliary stent retention. Endoscopic retrieval is the preferred method for forgotten biliary stents. However, long duration of retained stent, high stone burden, and altered anatomy often require surgical intervention [1-3]. CBD exploration followed by choledochoduodenostomy is a reliable option for permanent biliary drainage in patients with dilated CBD and stentolith formation [7,8]. In our patient, open CBD exploration allowed complete stentolith removal followed by side-to-side choledochoduodenostomy to ensure adequate long-term biliary drainage. The present case demonstrates the spectrum of complications that can result from long-term retention of biliary stents. Most of these complications can be easily prevented by adequate patient education and timely removal of stents [9-11].

## Conclusions

Prolonged retention of CBD stents is a rare condition associated with various complications like recurrent cholangitis, stentolith formation, and secondary biliary cirrhosis. All these can easily be prevented by adequate patient education regarding the importance of follow-up visits and timely removal of the stent. Retention of a CBD stent for 23 years highlights various issues like a lack of clear documentation at the time of stent placement, poor communication, and the importance of stent removal despite complete symptom relief.
